# Q-GrAM: Fine-Grained Image–Text Retrieval via Grouped Query Routing and Conditional Query Modulation

**DOI:** 10.3390/s26134313

**Published:** 2026-07-07

**Authors:** Guihe Gu, Huawei Li, Hong Qin

**Affiliations:** 1 School of Computer Science, Wuhan University, Wuhan 430072, China; 2 School of Artificial Intelligence, Wuhan University, Wuhan 430072, China

**Keywords:** image–text retrieval, fine-grained retrieval, text-to-image retrieval, grouped query routing, group-aware late interaction, Q-Former

## Abstract

Existing image–text retrieval methods often compute cross-modal similarity using global single-vector representations. Although efficient for coarse semantic alignment, such compressed representations are limited when textual queries involve fine-grained semantics, including objects, attributes, relations, and their compositional structures. This paper focuses on fine-grained text-to-image retrieval and proposes Q-GrAM, a retrieval-oriented adaptation of the BLIP-2 Q-Former. Instead of treating Q-Former queries as a homogeneous set, Q-GrAM partitions a fixed query budget into semantically differentiated groups. A text-guided router assigns token-level semantic demands to query groups, while query conditional initialization modulates each group according to group-level textual summaries. The resulting grouped visual query features are matched with text tokens through a group-aware late interaction scorer, and auxiliary routing balance and inter-group diversity regularization are introduced to stabilize semantic specialization. Experiments on MS-COCO 5K, Flickr30K, and Flickr30K-CFQ show that Q-GrAM achieves strong text-to-image retrieval performance against both global embedding baselines and representative fine-grained image–text matching methods, while maintaining competitive bidirectional retrieval performance. These results demonstrate the effectiveness of structured, text-conditioned Q-Former query specialization for fine-grained text-driven image search.

## 1. Introduction

Image text retrieval aims to learn a shared cross-modal representation space for bidirectional matching between images and natural language, and it remains a fundamental problem in vision–language understanding. In this work, we focus on fine-grained text-to-image retrieval, where a natural-language query is used to retrieve visually and semantically matched images from a candidate gallery. Recent advances in large-scale contrastive learning have substantially improved global semantic alignment, leading to strong progress on standard retrieval benchmarks. Nevertheless, many efficient retrieval systems and vision–language pretraining baselines still rely on global single-vector representations or highly compressed matching scores at inference time. Such formulations are often inadequate for complex textual queries that involve fine-grained semantics, including objects, attributes, relations, and their compositional dependencies. As a consequence, retrieval systems may produce semantically plausible yet incomplete matches when multiple candidate images share similar global semantics but differ in the local details most relevant to the query. This limitation has also been emphasized in prior work on fine-grained vision–language pretraining, which shows that global matching can overlook important correspondences between local image regions and word-level textual units [1,2].

The need for fine-grained matching is also evident in practical retrieval scenarios where user intent is often underspecified, evolving, or only partially expressed by the initial query. Recent interactive retrieval systems have shown that user feedback, iterative refinement, and semantic query rewriting can improve retrieval adaptability in multiturn search [3,4,5,6,7]. Such systems typically update query representations, adjust ranking scores, or refine textual descriptions according to user-provided relevance signals. However, their effectiveness is ultimately constrained by the discriminative capacity of the underlying retrieval backbone. If the backbone cannot reliably distinguish subtle object attributes, spatial relations, or compositional visual details, feedback-driven refinement may repeatedly operate on coarse or ambiguous matching evidence. In this case, interactive refinement can only partially compensate for errors introduced by weak visual–textual alignment. Therefore, improving the fine-grained matching capability of the retrieval model itself remains essential for robust text-driven image search.

Addressing the above limitation requires both stronger fine-grained matching and more flexible visual feature extraction. Late interaction modeling has emerged as an effective way to enhance fine-grained alignment by retaining token-level representations during matching. In information retrieval, ColBERT shows that token-level similarity aggregation can achieve a favorable balance between retrieval accuracy and computational efficiency [8]. In vision–language learning, FILIP extends this idea to cross-modal pretraining by introducing token-wise maximum similarity between textual words and image patches, thereby strengthening fine-grained alignment beyond global pooling [1]. More recent work such as SPARC further explores sparse fine-grained alignment between textual tokens and grouped visual patches, confirming the importance of local semantic structure in image–text representation learning [2]. Beyond late interaction, recent fine-grained image–text matching methods have also introduced more structured alignment mechanisms. CHAN improves local cross-modal matching through hard fragment alignment, HREM models semantic relations at both fragment and instance levels, and CORA composes object, attribute, and relation information through caption scene graphs [9,10,11]. These methods demonstrate that fine-grained semantic structure is crucial for accurate image–text matching, but their designs mainly focus on region–word alignment, relation reasoning, or text-side semantic composition.

In parallel, BLIP-2 introduces the Q-Former as a lightweight query interface that extracts a fixed number of informative visual features from a frozen image encoder [12]. This design provides a flexible bridge between visual representations and downstream language processing, and also suggests that learnable query tokens can serve as compact carriers of visual semantics. InstructBLIP further indicates that query-based visual extraction can be conditioned on textual input [13]. However, the internal query structure of Q-Former-based visual extraction remains underexplored for fine-grained retrieval. Existing fine-grained matching methods typically introduce structure through region–word interaction, relation modeling, or semantic graph composition, while Q-Former-based models usually treat learnable queries as a homogeneous set rather than semantically differentiated visual matching units. This gap motivates us to investigate whether Q-Former queries can be explicitly organized, conditioned, and matched as text-routed semantic groups for fine-grained text-driven image search.

Motivated by this gap, we propose Q-GrAM, a retrieval-oriented query specialization method built upon the BLIP-2 Q-Former for fine-grained text-to-image retrieval. Instead of treating Q-Former queries as a homogeneous set of visual tokens, Q-GrAM partitions the learnable queries into multiple groups while keeping the total query budget unchanged. A text-guided routing module predicts token-to-group relevance weights, enabling different query groups to focus on complementary semantic factors expressed in the text query. To further adapt visual feature extraction to query-specific semantics, we introduce Query Conditional Initialization, which uses group-level textual summaries to modulate the initialization of each query group. At the matching stage, we develop a group-aware late interaction function that aggregates group-wise maximum similarities under token-to-group routing weights, thereby supporting structured token-level visual–textual alignment. During training, we optimize the model with a bidirectional contrastive retrieval objective, together with routing balance and inter-group diversity constraints to mitigate routing collapse and functional redundancy across query groups. In this way, Q-GrAM combines text-conditioned query specialization with late interaction matching, improving the fine-grained discriminative capacity of Q-Former-based retrieval.

The main contributions of this work are summarized as follows.

- We propose Q-GrAM, a retrieval-oriented query specialization method for fine-grained text-to-image retrieval. It introduces grouped query routing under a fixed Q-Former query budget, enabling semantically differentiated visual query groups for structured feature extraction.
- We develop Query Conditional Initialization and a group-aware late interaction matching mechanism. These components adapt grouped queries to textual semantics and aggregate group-wise token similarities under routing guidance, thereby strengthening token-level visual–textual alignment.
- We design a training objective that combines contrastive retrieval, routing balance, and inter-group diversity constraints. Experiments on MS-COCO 5K, Flickr30K, and Flickr30K-CFQ against both global embedding baselines and representative fine-grained image–text matching methods show that Q-GrAM achieves strong text-to-image retrieval performance while maintaining competitive bidirectional retrieval behavior on standard caption-retrieval benchmarks.

## 2. Related Work

### 2.1. Image–Text Retrieval and Vision–Language Pretraining

Image–text retrieval aims to measure the semantic correspondence between visual content and natural language. Early visual-semantic embedding methods typically learn a joint representation space where paired images and texts are pulled together and mismatched pairs are pushed apart. With the emergence of large-scale vision–language pretraining, contrastive dual-encoder models have become a dominant paradigm for efficient retrieval. CLIP demonstrates that large-scale image–text contrastive learning can produce transferable visual representations, while ALIGN further shows that retrieval performance can benefit from scaling both model capacity and noisy web-supervised data [14,15,16,17]. Subsequent methods introduce stronger multimodal modeling and improved training objectives. ALBEF combines image–text contrastive learning with multimodal fusion through an align-before-fuse strategy, and BLIP unifies understanding and generation objectives for vision–language pretraining [18,19,20,21]. SigLIP further revisits the contrastive objective by replacing the softmax loss with a sigmoid loss, improving large-scale image–text representation learning [22].

The semantic capacity of CLIP-style representations has also been explored beyond standard retrieval. For example, StyleCLIP shows that CLIP can provide semantic directions for text-driven image manipulation, suggesting that language–image representations encode fine-grained semantic factors [23]. CLIPCEIL further adapts CLIP through channel refinement and image–text alignment to improve domain generalization under distribution shifts [24]. These studies support the importance of rich cross-modal semantic alignment, but they are not specifically designed for fine-grained text-to-image retrieval. In standard retrieval pipelines, many models still rely on global single-vector representations or highly compressed matching scores at inference time. Such representations are efficient, but they can be insufficient when a textual query requires discrimination among subtle object attributes, spatial relations, or compositional visual details. This motivates retrieval models that preserve richer local or token-level matching signals.

### 2.2. Fine-Grained Image–Text Matching and Late Interaction

Fine-grained image–text matching has been widely studied to overcome the limitations of global similarity computation. A major line of work focuses on region–word alignment. SCAN introduces stacked cross attention to infer latent alignments between salient image regions and sentence words [25]. IMRAM further performs iterative matching with recurrent attention memory, progressively refining cross-modal correspondences [26]. Later methods improve local matching reliability by filtering noisy fragments or reasoning over alignment structures. BFAN introduces bidirectional focal attention to suppress irrelevant fragments during cross-modal matching, SGRAF combines similarity graph reasoning with attention filtration to exploit both global and local alignments, and NAAF introduces negative-aware attention to explicitly model the effects of mismatched fragments [27,28,29]. Another group of methods strengthens fine-grained matching through relation reasoning, message passing, and graph-structured semantic modeling. VSRN performs visual semantic reasoning over image regions with graph-based relation modeling, while CAMP adaptively passes messages across visual and textual modalities before computing the final matching score [30,31]. GSMN explicitly constructs graph structures over objects, relations, and attributes, and performs node-level and structure-level matching to learn fine-grained phrase correspondence [32]. More recent methods further improve structured matching. CHAN formulates fine-grained matching through cross-modal hard alignment, HREM models semantic relationships at both fragment and instance levels, and CORA composes object, attribute, and relation information through caption scene graphs for efficient image–text matching [9,10,11].

Another related line of work improves fine-grained matching through late interaction. In information retrieval, ColBERT represents queries and documents with contextualized token embeddings and computes relevance through token-level late interaction, achieving a favorable trade-off between effectiveness and efficiency [8]. In vision–language learning, FILIP extends token-wise interaction to image–text pretraining by computing maximum similarities between textual tokens and visual patches [1]. SPARC further explores sparse fine-grained alignment between textual tokens and grouped visual patches, showing that preserving local semantic structure benefits image–text representation learning [2]. VL-Match further enhances vision–language pretraining with token-level and instance-level matching objectives, including token-level replaced-token detection and fine-grained instance-level image–text matching [33]. These methods demonstrate that token-level, instance-level, or fragment-level alignment is critical for fine-grained retrieval. However, they mainly introduce structure through region–word attention, fragment filtering, relation reasoning, scene-graph composition, or patch–token interaction. They do not explicitly investigate how the learnable query tokens of a Q-Former should be organized and matched for retrieval-oriented fine-grained visual extraction.

### 2.3. Query-Based Visual Extraction and Retrieval-Oriented Query Specialization

Query-based visual extraction has become an important design in modern vision–language models. BLIP-2 introduces the Q-Former as a lightweight trainable module that bridges a frozen image encoder and a frozen large language model [12]. The Q-Former uses a fixed set of learnable query tokens to extract compact visual representations from frozen image features, providing an efficient interface between visual encoders and language models. InstructBLIP further extends this idea by making visual extraction instruction-aware, allowing textual instructions to interact with query embeddings and improve task-relevant visual feature extraction [13]. These works show that learnable queries can serve as compact carriers of visual semantics and that query-based extraction can be conditioned on language.

Despite their effectiveness, existing Q-Former-based models usually treat learnable queries as a homogeneous set of visual tokens. Their query structure is primarily used to compress visual information for downstream multimodal reasoning or generation, rather than to provide semantically differentiated matching units for fine-grained retrieval. In contrast, Q-GrAM focuses on retrieval-oriented query specialization. Instead of increasing the number of queries, it partitions a fixed Q-Former query budget into multiple text-routed groups, modulates each group according to textual semantics, and performs group-aware late interaction over the resulting query representations. This design differs from prior fine-grained matching methods that rely on explicit region–word alignment, relation modeling, or scene-graph construction. It investigates the internal organization of Q-Former queries as a source of structured fine-grained visual–textual matching for text-driven image search.

## 3. Methods

We propose a fine-grained text-to-image retrieval method based on grouped query routing and group-aware late interaction matching. The method is built upon the Q-Former in BLIP-2 and performs grouped modeling over learnable query tokens under a fixed query budget. It further introduces text-driven group-level routing and conditional modulation to enhance fine-grained semantic alignment for complex textual queries. In BLIP-2, the Q-Former uses learnable queries to extract a fixed number of visual features from a frozen image encoder, which provides the structural basis for our grouped query design [12].

Inspired by routing mechanisms in mixture-of-experts models, we route the semantic demands of text tokens to different query groups and impose a load-balancing constraint to stabilize inter-group specialization. We emphasize that our method does not introduce sparse expert computation. Instead, it restructures query organization and the matching process under shared Q-Former parameters to improve fine-grained retrieval matching.

### 3.1. Overview and Notation

Given an image *I* and a text query *q*, our primary goal is to learn a retrieval scoring function S(q,I) for text-to-image ranking. We also use a symmetric contrastive objective during training and report image-to-text retrieval for completeness.

Let the frozen image encoder produce image features

(1) $$X\in R^{N\times d_{v}},$$

where *N* is the number of image tokens and $d_{v}$ is the image feature dimension.

Let the text encoder output token-level representations

(2) $$E=\left[e_{1},e_{2},\ldots ,e_{L}\right],\;e_{i}\in R^{d_{t}},$$

where *L* is the number of text tokens and $d_{t}$ is the text feature dimension.

On the Q-Former side, let the learnable query parameters be

(3) $$Q_{0}\in R^{K\times d_{q}},$$

where *K* is the total number of queries and $d_{q}$ is the query dimension. We partition the *K* queries into *G* query groups while keeping the total query budget unchanged. Let

(4) k=K/G,

with the divisibility constraint KmodG=0. The *j*th query group is denoted by

(5) $$Q_{0}^{\left(j\right)}\in R^{k\times d_{q}},\;j\in \left\{1,\ldots ,G\right\}.$$

**Figure 1:**
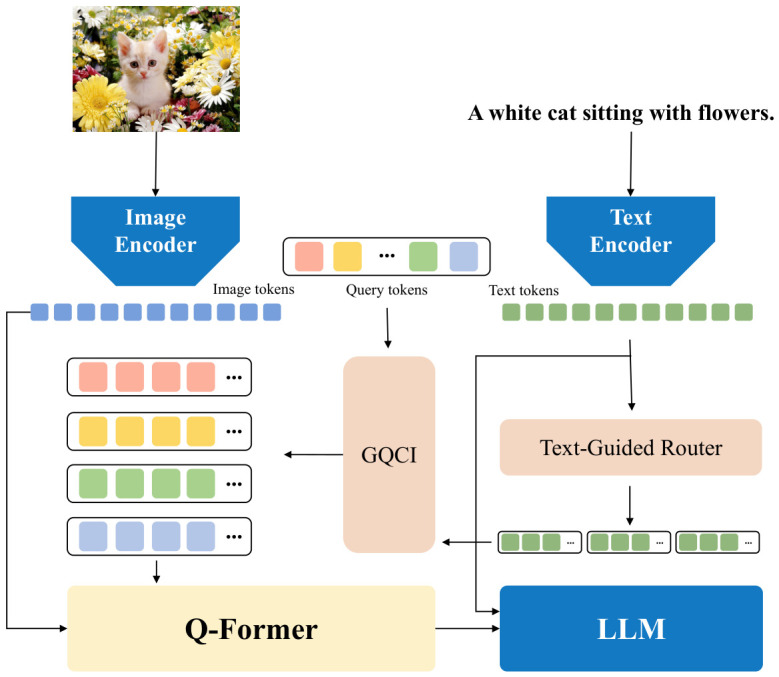
Overview of the proposed Q-GrAM framework. Given image and text inputs, the model first encodes visual and textual tokens, then performs text-guided query grouping and Query Conditional Initialization, followed by grouped visual feature extraction with the Q-Former. Finally, a group-aware late interaction module computes the retrieval score through routing-weighted similarity aggregation.

Based on the above definitions, the proposed method consists of grouped query routing, group-level conditional modulation with visual feature extraction, and group-aware late interaction matching. The central idea is to enhance fine-grained alignment under complex semantic queries by introducing text-driven query group specialization and structured matching within a fixed query budget. The overall pipeline is shown in Figure 1.

### 3.2. Grouped Query Routing

To encourage complementary representations across query groups over semantic dimensions such as objects, attributes, relations, and global context, we introduce a text-driven group-level routing mechanism. This mechanism maps the semantic demands of text tokens to multiple query groups, thereby avoiding a uniform interaction pattern in which all text tokens interact with all queries in the same manner.

For each text token representation $e_{i}$, the router outputs a weight distribution over *G* query groups:

(6) $$r_{i}=\mathrm{softmax}\left(f_{\mathrm{router}}\left(e_{i}\right)\right)\in R^{G},$$

where $f_{\mathrm{router}}\left(\cdot \right)$ is a lightweight MLP, and $r_{i,j}$ denotes the routing weight from the *i*th text token to the *j*th query group. By stacking the routing outputs of all text tokens, we obtain

(7) $$R\in R^{L\times G}.$$

Soft routing is adopted instead of hard assignment to preserve end-to-end differentiability and improve optimization stability. In implementation, we use top-*k* routing with k=2. For each token, only the two largest routing weights are retained, and the retained weights are renormalized before group-aware matching. This preserves differentiable soft weighting while encouraging each token to interact with a small subset of relevant query groups.

Based on the routing weights, we further construct group-level textual summaries as conditional inputs for the subsequent query modulation module:

(8) $$g_{j}=\frac{\sum_{i=1}^{L}r_{i,j}\;\phi \left(e_{i}\right)}{\sum_{i=1}^{L}r_{i,j}+\epsilon },\;g_{j}\in R^{d_{g}},$$

where ϕ(·) is a learnable projection layer, $d_{g}$ is the group summary dimension, and ϵ is a numerical stability term.

### 3.3. Group-Level Conditional Modulation and Feature Extraction

#### 3.3.1. Group-Level Conditional Modulation

To enable each query group to adaptively adjust its feature extraction preference according to the semantic requirements of the current text query, we generate group-specific modulation parameters from the group-level textual summary $g_{j}$. Specifically, a hidden representation is first computed via a feed-forward network:

(9) $$h_{j}=\mathrm{GELU}\left(W_{1}g_{j}+b_{1}\right).$$

A gating parameter $\alpha^{\left(j\right)}$ can be further generated from $h_{j}$ and combined with the offset term:

(10) $$\alpha^{\left(j\right)}=\sigma \left(W_{\alpha }h_{j}+b_{\alpha }\right).$$

Here, $\alpha^{\left(j\right)}$ may be defined as a scalar gate for each query or extended to a dimension-wise gate. The final query representation is

(11) $$Q^{\left(j\right)}=\alpha^{\left(j\right)}⊙Q_{0}^{\left(j\right)}+\beta \Delta Q^{\left(j\right)},$$

where ⊙ denotes element-wise multiplication. The gating term modulates the activation strength of queries within each group, while the offset term provides text-dependent directional correction. In our implementation, offset modulation is used as the default setting.

#### 3.3.2. Feature Extraction with Grouped Queries

The modulated query groups ${\left\{Q^{\left(j\right)}\right\}}_{j=1}^{G}$, together with the frozen image encoder output *X*, are fed into the Q-Former to extract group-level visual features. In practice, all query groups share the same Q-Former parameters and can be processed by query-dimension concatenation or batch-dimension expansion.

Let the output of the *j*th query group be

(12) $$Z^{\left(j\right)}=\left[z_{1}^{\left(j\right)},z_{2}^{\left(j\right)},\ldots ,z_{k}^{\left(j\right)}\right]\in R^{k\times d_{q}}.$$

By concatenating all group outputs along the query dimension, we obtain

(13) $$Z=\left[Z^{\left(1\right)};Z^{\left(2\right)};\ldots ;Z^{\left(G\right)}\right]\in R^{K\times d_{q}}.$$

The concatenated features are then projected into the retrieval embedding space:

(14) $$V=ZW_{v}\in R^{K\times d},$$

where $W_{v}\in R^{d_{q}\times d}$ and *d* denotes the retrieval embedding dimension. Correspondingly, the text token representations are projected into the same space:

(15) $$T=EW_{t}\in R^{L\times d}.$$

To improve numerical stability in similarity computation, $L_{2}$ normalization is applied to both *V* and *T*.

### 3.4. Group-Aware Late Interaction Matching

To achieve fine-grained visual–textual alignment, we construct a group-aware late interaction similarity function. Unlike standard late interaction methods that apply maximum similarity aggregation uniformly over all visual tokens, the proposed formulation introduces query-group structure and routing weights at the matching stage, enabling each text token to preferentially match query groups with higher semantic relevance.

Let $t_{i}$ denote the projected representation of the *i*th text token, and let $Q_{j}$ denote the index set of visual tokens corresponding to the *j*th query group. We first define the intra-group maximum similarity between the *i*th text token and the *j*th query group as

(16) $$s_{i,j}=\max_{m\in Q_{j}}\cos\left(t_{i},v_{m}\right).$$

The group-wise similarities are then aggregated using routing weights:

(17) $$s_{i}=\sum_{j=1}^{G}r_{i,j}s_{i,j}.$$

Finally, the scores of valid text tokens are aggregated to obtain the image–text matching score:

(18) $$S\left(q,I\right)=\frac{1}{L^{\prime }}\sum_{i\in I_{\mathrm{valid}}}s_{i},$$

where $I_{\mathrm{valid}}$ denotes the set of valid text token indices participating in matching and $L^{\prime }=\left|I_{\mathrm{valid}}\right|$. In implementation, padding tokens and special tokens are removed from the valid token set before score aggregation.

The essential characteristic of this matching formulation is that text tokens no longer match all visual tokens with equal weighting. Instead, routing weights explicitly introduce structured semantic bias at the matching stage, thereby improving discriminative capability under complex semantic queries.

### 3.5. Training Objective

The proposed method uses a bidirectional contrastive retrieval loss as the primary optimization objective, together with an inter-group diversity constraint and a routing load-balancing constraint, in order to improve retrieval performance and mitigate routing collapse as well as functional redundancy across query groups.

#### 3.5.1. Bidirectional Contrastive Retrieval Loss

Let the batch size be *B*, and let the *b*th sample be denoted by $\left(q_{b},I_{b}\right)$. A symmetric contrastive learning objective is constructed using in-batch negatives. The text-to-image loss is defined as

(19) $$L_{t2i}=-\frac{1}{B}\sum_{b=1}^{B}\log\frac{\exp(S\left(q_{b},I_{b}\right)/\tau )}{\sum_{b^{\prime }=1}^{B}\exp\left(S\left(q_{b},I_{b^{\prime }}\right)/\tau \right)},$$

and the image-to-text loss is defined as

(20) $$L_{i2t}=-\frac{1}{B}\sum_{b=1}^{B}\log\frac{\exp(S\left(q_{b},I_{b}\right)/\tau )}{\sum_{b^{\prime }=1}^{B}\exp\left(S\left(q_{b^{\prime }},I_{b}\right)/\tau \right)},$$

where τ is the temperature parameter. The main retrieval loss is

(21) $$L_{ret}=L_{t2i}+L_{i2t}.$$

#### 3.5.2. Inter-Group Diversity Constraint

To reduce the risk that different query groups learn highly redundant visual representations, we apply average pooling within each group:

(22) $$u_{j}=\frac{1}{k}\sum_{m\in Q_{j}}v_{m}.$$

The inter-group diversity constraint is defined as

(23) $$L_{div}=\sum_{j\neq j^{\prime }}\cos{\left(u_{j},u_{j^{\prime }}\right)}^{2}.$$

This term encourages complementary feature learning across groups by penalizing excessively high similarity between group representations.

#### 3.5.3. Routing Load-Balancing Constraint

Motivated by auxiliary load-balancing loss designs in MoE routing training, we introduce a group-level load-balancing constraint to prevent the router from assigning a disproportionate number of text tokens to a small subset of query groups. The differentiable auxiliary load-balancing loss used in Switch Transformer provides methodological support for this design [34].

Let the average group usage within a batch be

(24) $${\bar{p}}_{j}=\frac{1}{BL}\sum_{b=1}^{B}\sum_{i=1}^{L}r_{i,j}^{\left(b\right)}.$$

To encourage group usage to approach the uniform distribution 1/G, we define

(25) $$L_{bal}=\sum_{j=1}^{G}{\left({\bar{p}}_{j}-\frac{1}{G}\right)}^{2}.$$

This constraint helps mitigate routing collapse and improves the stability of inter-group specialization.

#### 3.5.4. Overall Objective

The final training objective is defined as

(26) $$L=L_{ret}+\lambda_{div}L_{div}+\lambda_{bal}L_{bal},$$

where $\lambda_{div}$ and $\lambda_{bal}$ are weighting coefficients, typically set to small values so as to avoid excessively perturbing the primary retrieval objective.

## 4. Application Scenario: Interactive Retrieval with Q-GrAM

We present *GrAM-IRIS*, an interactive image–text retrieval system that operationalizes Q-GrAM for intent-sensitive search over large-scale image collections. GrAM-IRIS is designed for practical retrieval scenarios where user intent may be underspecified, compositional, or progressively clarified through interaction. Rather than treating retrieval as a one-shot ranking problem, the system supports iterative intent steering by combining Q-GrAM scoring with lightweight user feedback signals.

### 4.1. System Architecture

**Figure 2:**
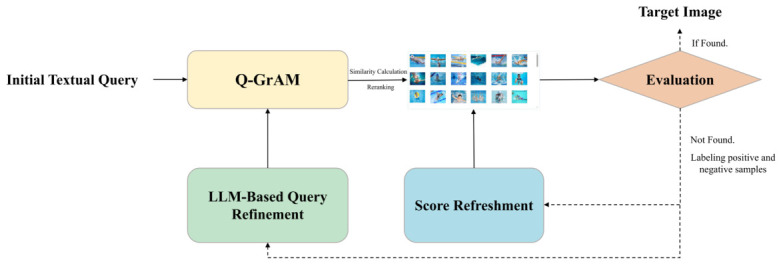
Overview of the GrAM-IRIS interactive retrieval system. The system performs initial retrieval with Q-GrAM and supports iterative intent steering via user feedback and stateful reranking.

Figure 2 provides an overview of the pipeline. Given an initial text query, the system computes an initial ranking using Q-GrAM and presents the top results to the user. The user may optionally provide feedback by marking returned images as relevant or irrelevant. GrAM-IRIS maintains a retrieval state that aggregates the original query evidence and the feedback evidence. The gallery is then reranked under the updated state, and the loop repeats until the returned results match the user intent.

### 4.2. Interaction Interface and Feedback Signals

**Figure 3:**
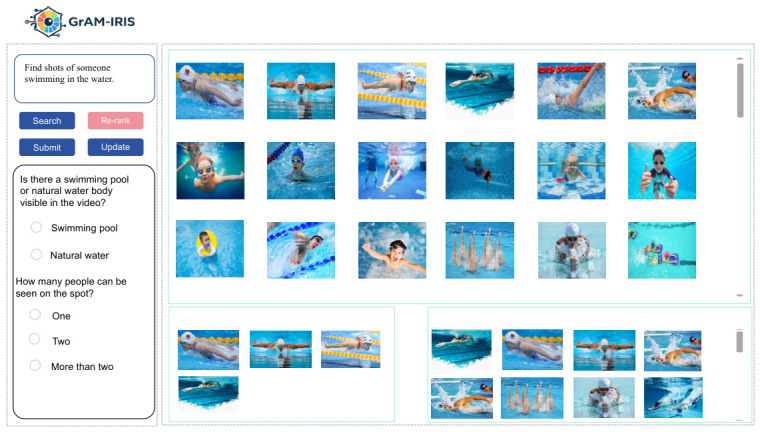
User interface of GrAM-IRIS. Users issue a text query, browse ranked results, and refine retrieval through positive and negative feedback.

GrAM-IRIS exposes a unified interface for text-to-image retrieval and interactive refinement (Figure 3). Users submit a natural language query, inspect ranked results, and optionally provide feedback by selecting positive and negative examples. This feedback is interpreted as a supervision signal on the current ranking, enabling the system to adjust retrieval behavior without requiring users to rewrite the query manually.

### 4.3. Retrieval Engine with Q-GrAM

Q-GrAM serves as the core scoring engine in GrAM-IRIS. For a query *q* and an image *I*, Q-GrAM computes a fine-grained similarity score S(q,I) by combining grouped query routing, query conditional initialization, and group-aware late interaction matching. In practice, this structured scoring function is advantageous for interactive retrieval because it provides token-level evidence that can be reused when updating the retrieval state.

### 4.4. Stateful Reranking for Intent Steering

To incorporate user feedback, GrAM-IRIS maintains a stateful scoring function that blends the original query signal with feedback-derived signals. Let ${\mathrm{Score}}_{\mathrm{prev}}\left(I\right)$ denote the score used in the previous iteration. After collecting a set of positive samples P and negative samples N from user feedback, the system updates the score by a convex combination:

(27) $${\mathrm{Score}}_{\mathrm{new}}\left(I\right)=\left(1-\kappa \right)\;{\mathrm{Score}}_{\mathrm{prev}}\left(I\right)+\kappa \;\Delta \left(I;P,N\right),$$

where κ∈(0,1) controls the update strength and Δ(·) summarizes the feedback-induced adjustment.

In our implementation, Δ(I;P,N) can be instantiated in a purely score-based manner using similarity statistics to positives and negatives, enabling efficient reranking without modifying the original query text. This stateful update strategy allows the system to progressively steer retrieval toward the desired intent with minimal interaction overhead.

## 5. Results

This section reports the empirical evaluation of the proposed method. We first describe the experimental setup and implementation details, and then present the main comparison results and ablation studies.

### 5.1. Experimental Setup

We implement Q-GrAM by modifying BLIP-2 OPT-2.7B with grouped query routing, Query Conditional Initialization, and group-aware late interaction matching. The model is trained using the COCO2014 training split only. Evaluation is conducted under a no-adaptation protocol. Specifically, the trained model is evaluated on the standard MS-COCO 5K test split without additional tuning. For cross-dataset evaluation, the same model is directly evaluated on Flickr30K without using any Flickr30K training data.

We evaluate both text-to-image retrieval and image-to-text retrieval using recall at K, including R@1, R@5, and R@10. Following common image–text retrieval practice, we also report the average recall for each retrieval direction and the overall average across both directions. Because the primary application scenario of Q-GrAM is text-driven image search, we pay particular attention to text-to-image retrieval, while also reporting image-to-text retrieval for completeness. In addition to MS-COCO 5K and Flickr30K, we evaluate Text→Image retrieval on Flickr30K-CFQ [35]. Because Flickr30K-CFQ is designed for compact and fragmented text queries and does not define an Image→Text retrieval setting, we report Text→Image metrics only. Because each query may have multiple positive images, we distinguish between hit-based retrieval recall and fractional CFQ recall. Specifically, R@1, Hit@5, and Hit@10 follow the standard hit criterion, where a query is counted as correct if any positive image appears in the top-*k* retrieved results. CFQ-R@5 and CFQ-R@10 denote multipositive fractional recall, computed as hits/min(#positives,k). We also report MRR@10 and mAP@10 to evaluate ranking quality within the top-10 retrieved results.

We compare Q-GrAM with two groups of baselines. The first group includes global embedding baselines based on frozen encoders with a trainable projector, including CLIP and BLIP-2. The second group includes representative fine-grained image–text matching methods, including HREM, CHAN, and CORA. These methods cover hierarchical relation modeling, cross-modal hard alignment, and object-relation-attribute composition, respectively. Results of prior fine-grained methods are reported under the standard MS-COCO 5K and Flickr30K retrieval protocols, while our reproduced baselines and Q-GrAM are evaluated using the same metric definitions.

### 5.2. Implementation Details

We use BLIP-2 OPT-2.7B as the backbone model. Following the BLIP-2 design, the vision encoder and the large language model are kept frozen, while the Q-Former and the retrieval-specific modules are optimized. The trainable modules include the Q-Former, the language projection layer, the retrieval projection layers, the text-guided router, and the query modulation module.

All experiments are trained on the COCO2014 training split with bf16 mixed precision for 10 epochs. We use a per-step batch size of 128 without gradient accumulation, resulting in an effective batch size of 128. The maximum text length is set to 32. The learning rate is set to $1\times 10^{-4}$ with a weight decay of 0.01, and a linear warm-up schedule is used for the first 5% of the optimization steps. The contrastive temperature is set to 0.05.

For Q-GrAM, we use four query groups. The router hidden dimension is set to 256, and top-*k* routing is used with k=2. The router beta is initialized to 0.1 and gradually warmed up during early training. The weights of the inter-group diversity loss and the routing balance loss are both set to 0.02. Unless otherwise specified, the same hyperparameters are used for all ablation variants, with only the corresponding component removed. All experiments are conducted on an NVIDIA A100-PCIE-40GB GPU.

### 5.3. Main Comparison

Table 1 reports the main comparison on the standard MS-COCO 5K and Flickr30K caption-retrieval benchmarks. To further evaluate retrieval under more realistic query styles, Table 2 reports additional Text→Image results on Flickr30K-CFQ, a compact and fragmented query benchmark. Table 3 further reports paired bootstrap significance tests on Flickr30K-CFQ against representative fine-grained baselines.

It should be noted that Table 1 contains both reproduced baselines and reported prior results. CLIP and BLIP-2 are evaluated under our reproduced frozen-encoder-plus-projector protocol, whereas HREM, CHAN, and CORA are included as representative fine-grained image–text matching methods using results reported from their original publications. As these prior methods may differ in visual backbone, training data, model size, and implementation protocol, the comparison with † methods should be interpreted as a contextual comparison rather than a strictly controlled head-to-head evaluation. Accordingly, we use these results to position Q-GrAM with respect to major fine-grained matching paradigms, including hierarchical relation modeling, cross-modal hard alignment, and object-relation-attribute composition, while avoiding strong state-of-the-art claims based on unmatched protocols.

On MS-COCO 5K, Q-GrAM obtains the highest listed Text→Image retrieval scores among the results in Table 1. It obtains 49.38 R@1, 76.60 R@5, 85.44 R@10, and 70.47 T→I average. Compared with the reported CHAN result, which is the highest listed prior fine-grained baseline in the Text→Image direction, Q-GrAM improves T→I R@1 by 4.48 points and T→I average by 2.60 points. This result supports the effectiveness of text-guided grouped query routing for text-driven image search. In Image→Text retrieval, Q-GrAM does not outperform all prior fine-grained methods. HREM, CHAN, and CORA achieve stronger I→T averages, indicating that relation-based and compositional matching can be particularly effective for caption retrieval. Nevertheless, Q-GrAM remains competitive in the overall average and provides clear gains in the target Text→Image direction.

On Flickr30K, Q-GrAM also achieves the highest listed Text→Image retrieval scores among the listed methods, with 69.82 R@1, 90.84 R@5, 94.88 R@10, and 85.18 T→I average. Compared with the reported CORA result, Q-GrAM improves T→I R@1 by 5.72 points and T→I average by 3.41 points. For Image→Text retrieval, CORA achieves the highest I→T average, while Q-GrAM matches the highest listed I→T R@10 score. Overall, Q-GrAM obtains the highest listed average score on Flickr30K, showing that the proposed grouped query routing mechanism transfers effectively from COCO training to the standard Flickr30K caption-retrieval evaluation.

To examine whether Q-GrAM remains effective beyond standard caption-style retrieval, we further evaluate it on Flickr30K-CFQ. Unlike the original Flickr30K benchmark, Flickr30K-CFQ contains compact and fragmented textual queries and therefore better reflects realistic text-driven image search scenarios where user queries are often short, partial, or compositionally expressed. As Flickr30K-CFQ is designed for Text→Image retrieval, we report Text→Image metrics only, including hit-based recall, fractional CFQ recall, MRR@10, and mAP@10.

As shown in Table 2, Q-GrAM achieves the highest listed scores across all reported metrics among the representative fine-grained baselines on Flickr30K-CFQ. It obtains 41.33 R@1, 62.97 Hit@5, 72.12 Hit@10, 57.15 CFQ-R@5, 65.81 CFQ-R@10, 50.62 MRR@10, and 47.38 mAP@10. Compared with the strongest listed fine-grained baseline CHAN, Q-GrAM improves R@1 by 10.00 points and mAP@10 by 9.81 points. The improvement is especially notable on R@1 and mAP@10, indicating that text-routed query specialization helps Q-GrAM rank highly relevant images near the top of the retrieved list under compact and fragmented query expressions. These results provide additional evidence that the proposed grouped Q-Former query organization is useful not only for full-sentence caption retrieval, but also for more realistic fine-grained text-driven image search.

To assess whether the main gains over fine-grained baselines are robust at the query level, we further conduct paired bootstrap significance testing on Flickr30K-CFQ. We resample text queries with replacement for 10,000 bootstrap trials and compute the metric differences between Q-GrAM and representative fine-grained baselines. As shown in Table 3, Q-GrAM significantly improves over CHAN on both R@1 and mAP@10, with gains of 10.00 and 9.81 points, respectively, and both differences are significant with p<0.0001. The improvements over HREM and CoRA are also statistically significant. These results provide additional evidence that the proposed grouped Q-Former query organization is useful not only for full-sentence caption retrieval, but also for more realistic fine-grained text-driven image search.

Taken together, the results on standard caption-retrieval benchmarks and Flickr30K-CFQ suggest that Q-GrAM is particularly effective for fine-grained Text→Image retrieval. Unlike prior fine-grained methods that mainly rely on region–word hard alignment, hierarchical relation modeling, or scene-graph-based compositional encoding, Q-GrAM improves retrieval by reorganizing Q-Former visual queries into text-routed semantic groups under a fixed query budget. The consistent gains in the Text→Image direction, especially on compact and fragmented queries, are aligned with the design goal of Q-GrAM: strengthening text-conditioned visual feature extraction and structured matching for text-driven image search. At the same time, the Image→Text results on MS-COCO 5K and Flickr30K show that Q-GrAM is competitive rather than uniformly dominant in the reverse retrieval direction.

### 5.4. Ablation Studies

We conduct ablation studies to analyze the contribution of the main components in Q-GrAM. All variants are trained on the COCO2014 training split and evaluated on MS-COCO 5K and Flickr30K under the same no-adaptation protocol as the main comparison. The full model contains grouped query routing, Query Conditional Initialization, group-aware late interaction, routing balance regularization, and inter-group diversity regularization. The ablated variants remove one component at a time while keeping the remaining settings unchanged.

We organize the ablation analysis into three parts. First, Table 4 examines whether the gains of Q-GrAM come from simply increasing the number of Q-Former queries or from structured query utilization. Second, Table 5 and Table 6 analyze the contribution of individual Q-GrAM components. Third, Table 7 analyzes the learned routing behavior and the effect of the balance regularizer on preventing routing collapse. Overall, the results show that different components play different roles. Group-aware late interaction and text-guided routing are the main sources of retrieval improvement, Query Conditional Initialization mainly affects the balance between retrieval directions, and the balance and diversity losses behave primarily as structural regularizers rather than as uniformly performance-improving modules.

Table 4 shows that simply increasing the number of Q-Former queries is ineffective. The pooled Q-Former variants are ablation-specific configurations designed to isolate the effect of query number under pooled scoring. Therefore, Table 4 should be interpreted as a controlled comparison of query number, matching, grouping, and routing design choices within the ablation setting.

Increasing the query budget from 32 to 64 in the pooled Q-Former setting changes the overall average only from 61.06 to 61.03. This indicates that a larger query set does not automatically produce stronger retrieval representations, and that additional queries may remain redundant without an explicit specialization mechanism.

Introducing token-level late interaction brings a much larger improvement. The Late Interaction Q-Former improves the overall average from 61.06 to 71.00, confirming that token-query-level fine-grained matching is more effective than pooled global matching for text-driven image search. However, increasing the number of queries under the same late interaction setting again brings almost no benefit: the overall average remains nearly unchanged, from 71.00 to 70.99, while the COCO evaluation time increases from 291.2 s to 384.9 s. This result suggests that increasing query quantity is a less effective strategy than improving query utilization.

Uniform grouped late interaction produces no improvement over the ungrouped late interaction configuration. It obtains the same overall average as the Late Interaction Q-Former, indicating that grouping alone is insufficient when all groups are used uniformly. In contrast, text-routed grouped late interaction improves the overall average from 71.00 to 78.23 while still using only 32 queries. This is the key evidence that Q-GrAM benefits from structured, text-conditioned query utilization rather than from a larger query budget. The full Q-GrAM model further improves the overall average to 80.63, mainly through stronger Image→Text retrieval, while preserving the strong Text→Image performance obtained by text-routed grouped matching.

Removing group-aware late interaction leads to the most consistent degradation on both datasets. On MS-COCO 5K, the overall average decreases from 73.88 to 70.77, and on Flickr30K it decreases from 87.39 to 83.87. The degradation is particularly clear in the Text→Image direction, where the average recall drops from 70.47 to 65.81 on MS-COCO 5K and from 85.18 to 80.01 on Flickr30K. These results indicate that token-level and query-level fine-grained matching are critical for text-driven image search. Together with Table 4, these results indicate that the improvements in Q-GrAM do not come merely from using more Q-Former visual queries, but from using them through fine-grained late interaction and text-guided grouped routing.

The effect of Query Conditional Initialization is directional. Removing QCI improves Text→Image retrieval on both datasets, increasing the Text→Image average from 70.47 to 71.08 on MS-COCO 5K and from 85.18 to 85.47 on Flickr30K. This indicates that QCI is not the main source of the Text→Image gains and should not be interpreted as a component that uniformly improves the primary retrieval direction. However, removing QCI substantially reduces Image→Text performance, where the average drops from 77.29 to 74.03 on MS-COCO 5K and from 89.60 to 86.73 on Flickr30K. As a result, the overall average also decreases from 73.88 to 72.55 on MS-COCO 5K and from 87.39 to 86.10 on Flickr30K. We therefore interpret QCI as a bidirectional alignment stabilizer rather than a unidirectional Text→Image performance booster. In other words, the primary Text→Image improvements in Q-GrAM mainly come from grouped routing and group-aware late interaction, while QCI helps preserve more balanced cross-modal alignment across retrieval directions.

The routing balance loss has a limited effect on retrieval accuracy. Removing it produces nearly identical Text→Image performance on MS-COCO 5K and a slightly higher overall average on Flickr30K. Therefore, we do not interpret the balance loss as a component that consistently improves recall metrics. Its role is instead to regularize the routing distribution and discourage degenerate group usage during training. This is consistent with the design motivation of Q-GrAM, where query groups are expected to remain usable and semantically differentiated rather than collapsing to a small subset of dominant groups.

To verify this interpretation, we further analyze the routing behavior on a COCO validation subset containing 55,439 valid text tokens. As shown in Table 7, the full Q-GrAM model uses all four groups with a non-degenerate routing distribution. Its mean group mass is 18.4%, 22.5%, 38.6%, and 20.6%, and the effective number of used groups is 3.82 out of 4. Although the router shows a preference for the third group, the remaining groups still receive substantial routing mass, indicating that the learned routing does not collapse to a single group.

In contrast, removing the balance loss leads to severe routing collapse. Without the balance regularizer, 82.2% of the mean routing mass is assigned to one group, and the top-1 routing distribution assigns all valid tokens to the same group. The effective number of used groups drops from 3.82 to 1.83. This behavior explains why the balance loss is structurally important even when its effect on R@K metrics is modest. It prevents grouped routing from degenerating into single-group routing and helps preserve the intended multigroup specialization mechanism.

The diversity loss shows a similar regularization-oriented behavior, although its effect is not directly reflected by the routing-load statistics in Table 7. Removing it produces results close to the full model on MS-COCO 5K and slightly higher overall performance on Flickr30K. This indicates that inter-group diversity regularization is not the dominant source of retrieval improvement in the current setting. Nevertheless, it provides an explicit constraint for reducing functional redundancy among query groups and encouraging more interpretable group specialization. Therefore, we treat the diversity loss as a structural regularizer rather than as a direct accuracy-enhancing component.

In summary, the ablation results support three conclusions. First, simply increasing the number of Q-Former queries is ineffective: increasing the query budget from 32 to 64 does not improve retrieval performance under either global matching or ungrouped late interaction. Second, the main performance gains come from fine-grained late interaction and text-guided grouped routing, which substantially improve query utilization under a fixed query budget. Third, Query Conditional Initialization, balance loss, and diversity loss should be interpreted as auxiliary structural components rather than uniformly accuracy-improving modules. QCI introduces a trade-off by slightly reducing Text→Image recall while improving Image→Text retrieval and the overall bidirectional average. The balance loss is especially important for preventing routing collapse, as shown by the routing behavior analysis. These findings support the design of Q-GrAM as a structured query specialization method rather than a simple extension of the BLIP-2 Q-Former with more query tokens.

### 5.5. Efficiency Analysis

We further analyze the computational efficiency of Q-GrAM to examine whether the proposed grouped query routing and group-aware late interaction introduce substantial overhead. Table 8 reports the number of additional trainable parameters, encoding time, scoring time, total inference time, and peak GPU memory. Following the implementation protocol in our experiments, all measurements are conducted on an NVIDIA A100-PCIE-40 GB GPU.

Compared with the BLIP-2 baseline, Q-GrAM introduces a few additional parameters, with 3.51M trainable parameters. This indicates that the proposed grouped query routing and conditional modulation do not require a large parameter increase. In terms of runtime, Q-GrAM increases the total inference time from 209.96 ms to 242.08 ms, corresponding to an additional 32.12 ms, or approximately 15.3% relative overhead. The encoding time increases moderately from 209.87 ms to 224.87 ms, while the scoring time increases from 0.10 ms to 17.21 ms due to the group-aware late interaction computation.

Although the relative increase in scoring time is large because the baseline scoring operation is nearly negligible, the absolute scoring overhead remains moderate. Moreover, peak GPU memory remains almost unchanged, with 8.40 GB for Q-GrAM compared with 8.41 GB for the BLIP-2 baseline. These results suggest that Q-GrAM improves fine-grained text-to-image retrieval performance with acceptable inference overhead and without increasing peak memory consumption.

## 6. Conclusions

This paper presents Q-GrAM, a retrieval-oriented query specialization method built upon BLIP-2 OPT-2.7B for fine-grained text-to-image retrieval. Q-GrAM organizes the fixed Q-Former query budget into semantically differentiated groups, and combines text-guided routing, Query Conditional Initialization, and group-aware late interaction matching to strengthen structured visual–textual alignment. Extensive evaluations on MS-COCO 5K, Flickr30K, and Flickr30K-CFQ demonstrate that Q-GrAM achieves strong Text→Image retrieval performance compared with both global embedding baselines and representative fine-grained image–text matching methods. The bidirectional results further show that Q-GrAM remains competitive with relation-based and compositional prior methods, while exhibiting its strongest advantage in text-driven image search.

Ablation studies provide further insight into the contribution of each component. The results show that group-aware late interaction and text-guided grouped routing are the main sources of retrieval improvement, indicating the importance of structured token-level matching and text-conditioned query utilization. Query Conditional Initialization improves the balance of bidirectional alignment by adapting query groups to textual semantics, while the routing balance loss serves as a stabilizing regularizer that prevents routing collapse. Inter-group diversity regularization is better interpreted as an auxiliary structural constraint for reducing functional redundancy among query groups rather than as a uniformly accuracy-improving component. Overall, these findings suggest that explicitly organizing and routing Q-Former queries is an effective direction for improving fine-grained text-driven image retrieval under a fixed query budget.

## Figures and Tables

**Table 1:** Main comparison on MS-COCO 5K and Flickr30K. We report Text→Image retrieval, Image→Text retrieval, directional average recall, and the overall average. BLIP-2 and CLIP are evaluated as frozen encoder + projector baselines. HREM, CHAN, and CORA are representative prior fine-grained image–text matching methods. Results marked with † are reported from prior publications under the standard evaluation protocol. The best listed results are shown in bold, the second-best results are underlined, and the third-best results are double-underlined.

Method	Text→Image				Image→Text				Overall
	R@1	R@5	R@10	Avg	R@1	R@5	R@10	Avg	Avg
**MS-COCO 5K**									
BLIP-2	43.23	72.58	82.71	66.17	57.22	83.84	90.94	77.33	71.75
CLIP	39.95	67.94	78.75	62.21	55.06	80.42	88.28	74.59	68.40
HREM ^†^	44.00	73.70	83.40	67.03	61.80	87.00	93.20	**80.67**	73.85
CHAN ^†^	44.90	74.50	84.20	67.87	59.80	**87.30**	**93.30**	80.13	**74.00**
CORA ^†^	44.20	73.60	83.90	67.23	**62.40**	86.80	92.60	80.60	73.92
Q-GrAM	**49.38**	**76.60**	**85.44**	**70.47**	56.70	83.80	91.36	77.29	73.88
**Flickr30K**									
BLIP-2	58.16	85.00	91.02	78.06	75.50	93.80	97.50	88.93	83.50
CLIP	61.26	85.76	91.70	79.57	75.10	93.70	96.20	88.33	83.95
HREM ^†^	59.30	85.10	91.20	78.53	79.50	94.30	97.40	90.40	84.47
CHAN ^†^	55.60	81.20	87.50	74.77	68.70	91.50	95.30	85.17	79.97
CORA ^†^	64.10	88.10	93.10	81.77	**83.40**	**95.90**	**98.60**	**92.63**	87.20
Q-GrAM	**69.82**	**90.84**	**94.88**	**85.18**	75.20	95.00	**98.60**	89.60	**87.39**

**Table 2:** Text→Image retrieval results on Flickr30K-CFQ against representative fine-grained baselines. R@1, Hit@5, and Hit@10 follow the standard hit criterion, where a query is counted as correct if any positive image appears in the top-*k* retrieved results. CFQ-R@5 and CFQ-R@10 denote multipositive fractional recall, computed as hits/min(#positives,k). The highest listed values are shown in bold, and the second-highest listed values are underlined.

Method	R@1	Hit@5	Hit@10	CFQ-R@5	CFQ-R@10	MRR@10	mAP@10
HREM	20.97	43.21	53.15	37.98	46.87	30.29	27.14
CoRA	20.30	41.45	50.97	36.78	45.01	29.50	26.55
CHAN	31.33	54.67	63.03	48.63	55.91	41.13	37.57
Q-GrAM	**41.33**	**62.97**	**72.12**	**57.15**	**65.81**	**50.62**	**47.38**

**Table 3:** Paired bootstrap significance test against representative fine-grained baselines on Flickr30K-CFQ. We report the metric difference Δ = Q-GrAM − baseline, 95% confidence intervals, and two-sided bootstrap *p*-values over 10,000 query-level resamples.

Baseline	ΔR@1	95% CI	*p*	ΔmAP@10	95% CI	*p*
CHAN	+10.00	[7.57, 12.61]	<0.0001	+9.81	[7.83, 11.75]	<0.0001
HREM	+20.36	[17.88, 22.91]	<0.0001	+20.24	[18.20, 22.34]	<0.0001
CoRA	+21.03	[18.55, 23.64]	<0.0001	+20.83	[18.89, 22.81]	<0.0001

**Table 4:** Ablation study on query number, late interaction, grouping, and text-guided routing. All variants are trained on COCO2014 and evaluated on MS-COCO 5K and Flickr30K under the no-adaptation protocol. The pooled Q-Former variants are ablation-specific baselines with pooled scoring and are used for controlled comparison within this table. COCO Avg and Flickr Avg denote the average of Text→Image and Image→Text directional averages on each dataset. The best listed results are shown in bold, and the second-best results are underlined.

Variant	#Queries	Grouping	Routing	QCI	Matching	COCO Avg	Flickr Avg	Overall Avg
Pooled Q-Former	32	No	No	No	Global	53.18	68.94	61.06
Pooled Q-Former, Expanded	64	No	No	No	Global	53.05	69.01	61.03
Late Interaction Q-Former	32	No	No	No	Token max	63.94	78.06	71.00
Late Interaction Q-Former, Expanded	64	No	No	No	Token max	63.89	78.09	70.99
Uniform Grouped Late Interaction	32	Yes	Uniform	No	Group-aware	63.94	78.06	71.00
Text-Routed Grouped Late Interaction	32	Yes	Learned	No	Group-aware	71.15	85.32	78.23
Q-GrAM	32	Yes	Learned	Yes	Group-aware	**73.88**	**87.39**	**80.63**

**Table 5:** Ablation study on MS-COCO 5K. All variants are trained on the COCO2014 training split and evaluated under the no-adaptation protocol. The best listed results are shown in bold, and the second-best results are underlined.

	Text→Image				Image→Text				
Method	R@1	R@5	R@10	Avg	R@1	R@5	R@10	Avg	Overall Avg
Q-GrAM	49.38	76.60	85.44	70.47	**56.70**	**83.80**	**91.36**	**77.29**	**73.88**
w/o QCI	**50.56**	**77.01**	**85.67**	**71.08**	51.22	80.86	90.00	74.03	72.55
w/o Late Interaction	43.65	71.79	81.99	65.81	54.48	82.02	90.70	75.73	70.77
w/o Balance Loss	49.34	76.65	85.43	70.47	55.98	82.98	90.98	76.65	73.56
w/o Diversity Loss	49.54	76.65	85.42	70.54	55.82	83.20	91.34	76.79	73.66

**Table 6:** Ablation study on Flickr30K. All variants are trained on the COCO2014 training split and evaluated under the no-adaptation protocol. The best listed results are shown in bold, and the second-best results are underlined.

	Text→Image				Image→Text				
Method	R@1	R@5	R@10	Avg	R@1	R@5	R@10	Avg	Overall Avg
Q-GrAM	69.82	90.84	94.88	85.18	75.20	**95.00**	**98.60**	89.60	87.39
w/o QCI	**70.26**	**90.96**	**95.20**	**85.47**	70.10	92.50	97.60	86.73	86.10
w/o Late Interaction	62.34	85.82	91.86	80.01	73.90	93.30	96.00	87.73	83.87
w/o Balance Loss	69.36	90.44	94.84	84.88	**76.90**	94.80	98.30	**90.00**	87.44
w/o Diversity Loss	70.12	90.78	95.04	85.31	76.60	94.90	98.10	89.87	**87.59**

**Table 7:** Routing behavior analysis on the COCO validation subset. The analysis is computed over 55,439 valid text tokens. Mean group mass denotes the average final routing weight assigned to each group, and top-1 group distribution denotes the distribution of the highest-weight group for each token. Effective groups measures the effective number of groups used by the router.

Variant	Mean Group Mass (%)	Top-1 Group Distribution (%)	Max Mass (%)	Effective Groups	Collapse
Full Q-GrAM	[18.4, 22.5, 38.6, 20.6]	[8.6, 24.8, 50.8, 15.8]	38.6	3.82	No
w/o Balance Loss	[0.4, 7.5, 82.2, 10.0]	[0.0, 0.0, 100.0, 0.0]	82.2	1.83	Yes

**Table 8:** Efficiency analysis on an NVIDIA A100-PCIE-40GB GPU. BLIP-2 denotes the frozen BLIP-2 encoder with a trainable projector.

Method	Params Added	Encoding Time (ms)	Scoring Time (ms)	Inference Time (ms)	Peak Memory (GB)
BLIP-2	-	209.87	0.10	209.96	8.41
Q-GrAM	3.51M	224.87	17.21	242.08	8.40

## Data Availability

Publicly available datasets were analyzed in this study. The MS-COCO, Flickr30K, and Flickr30K-CFQ datasets used in this study are available from their respective official repositories or corresponding publications. No new dataset was created in this study.
